# A Randomized Blinded Study of the Left Ventricular Myocardial Performance Index Comparing Epinephrine to Levosimendan following Cardiopulmonary Bypass

**DOI:** 10.1371/journal.pone.0143315

**Published:** 2015-12-14

**Authors:** Marcello Fonseca Salgado Filho, Marselha Barral, Louis Barrucand, Ismar Lima Cavalcanti, Nubia Verçosa

**Affiliations:** 1 Federal University of Rio de Janeiro, Rio de Janeiro, Brazil; 2 Faculty of Medical Sciences of Juiz de Fora, Juiz de Fora, Brazil; 3 Fluminense Federal University, Niteroi, Brazil; Harefield Hospital, UNITED KINGDOM

## Abstract

**Background:**

The objective was to evaluate the effect of epinephrine and levosimendan on the left ventricle myocardial performance index in patients undergoing on-pump coronary artery by-pass grafting (CABG).

**Methods:**

In a double-blind, randomized clinical trial, 81 patients (age: 45–65 years) of both genders were randomly divided to receive either epinephrine at a dosage of 0.06 mcg.kg^1^.min^-1^ (epinephrine group, 39 patients) or levosimendan at 0.2 mcg.kg^1^.min^-1^ (levosimendan group, 42 patients) during the rewarming of cardiopulmonary by-pass (CPB). Hemodynamic data were collected 30 minutes after tracheal intubation, before chest open (pre-CPB) and 10 minutes after termination of protamine (post-CPB). As the primary outcome, we evaluated the left ventricle myocardial performance index by the Doppler echocardiography. The myocardial performance index is the sum of the isovolumetric contraction time and the isovolumetric relaxation time, divided by the ejection time. Secondary outcomes were systolic and diastolic evaluations of the left ventricle and postoperative troponin I and MB-CK levels.

**Results:**

Of the 81 patients allocated to the research, we excluded 2 patients in the epinephrine group and 6 patients in the levosimendan group because they didn’t wean from CPB in the first attempt. There was no statistical difference between the groups in terms of patient characteristics, risk factors, or CPB time. The epinephrine group had a lower left ventricle myocardial performance index (*p* = 0.0013), higher cardiac index (*p* = 0.03), lower systemic vascular resistance index (*p* = 0.01), and higher heart rate (*p* = 0.04) than the levosimendan group at the post-CPB period. There were no differences between the groups in diastolic dysfunction. The epinephrine group showed higher incidence of weaning from CPB in the first attempt (95% vs 85%, p = 0.0001) when compared to the levosimendan group and the norepinephrine requirement was higher in the levosimenandan group than epinephrine group (16% vs. 47%; p = 0.005) in post-CPB period. Twenty-four hours after surgery, the plasma levels of troponin I (epinephrine group: 4.5 ± 5.7 vs. levosimendan group: 2.5 ± 3.2 g/dl; p = 0.09) and MB-CK (epinephrine group: 50.7 ± 31 vs. levosimendan group: 37 ± 17.6 g/dl; p = 0.08) were not significantly different between the two groups.

**Conclusion:**

When compared to levosimendan, patients treated with epinephrine had a lower left ventricle myocardial performance index in the immediate post-CPB period, encouraging an efficient weaning from CPB in patients undergoing on-pump CABG.

**Trial Registration:**

ClinicalTrials.gov NCT01616069

## Introduction

Myocardial ischemia provoked by coronary artery disease and inflammatory changes related to coronary artery bypass graft (CABG) surgery with cardiopulmonary bypass (CPB) can lead to systolic and diastolic dysfunction of the left ventricle and impair patient outcome [1].

Myocardial function can be impaired by aortic cross clamping, lesions in the coronary microcirculation [2], cardioplegic arrest and postischemic stunning [3], even in patients with normal ejection fraction underwent on-pump CABG [4–6]. Therefore, weaning from CPB can be facilitated using cardiotonic drugs such as epinephrine and levosimendan. The inodilator levosimendan is a calcium sensitizer that increases myocardial contractility without excessively increasing intracellular calcium and oxygen consumption and shows myocardial protection effects [7, 8]. In contrast, epinephrine, a beta-receptor agonist, stimulates the production of cyclic adenosine monophosphate (cAMP), which increases intracellular calcium, myocardial contractility and oxygen consumption [9, 10].

The myocardial performance index (MPI), or the Tei index, measured by Doppler echocardiography, is related to the systolic (isovolumetric contraction time and ejection time) and diastolic (isovolumetric relation time) functions of the heart and is calculated as the sum of the isovolumetric contraction time and the isovolumetric relaxation time, divided by the ejection time [11–13]. The myocardial performance index is not related to ventricle geometry, heart rate or age [13–18].

There are questions regarding which is the better inotropic drug to use during the post-CPB period in patients undergoing on-pump CABG surgery. The hypothesis of the present study is that levosimendan without loading dose can reduce the left ventricle myocardial performance index and show a better myocardium protection than beta-adrenergic drugs such as epinephrine [19, 20].

The primary objective of the present study was to compare the effects of epinephrine and levosimendan on the left ventricle myocardial performance index in patients undergoing on-pump CABG surgery.

## Material and Methods

### Population study, protocol, and randomization

**Fig 1:**
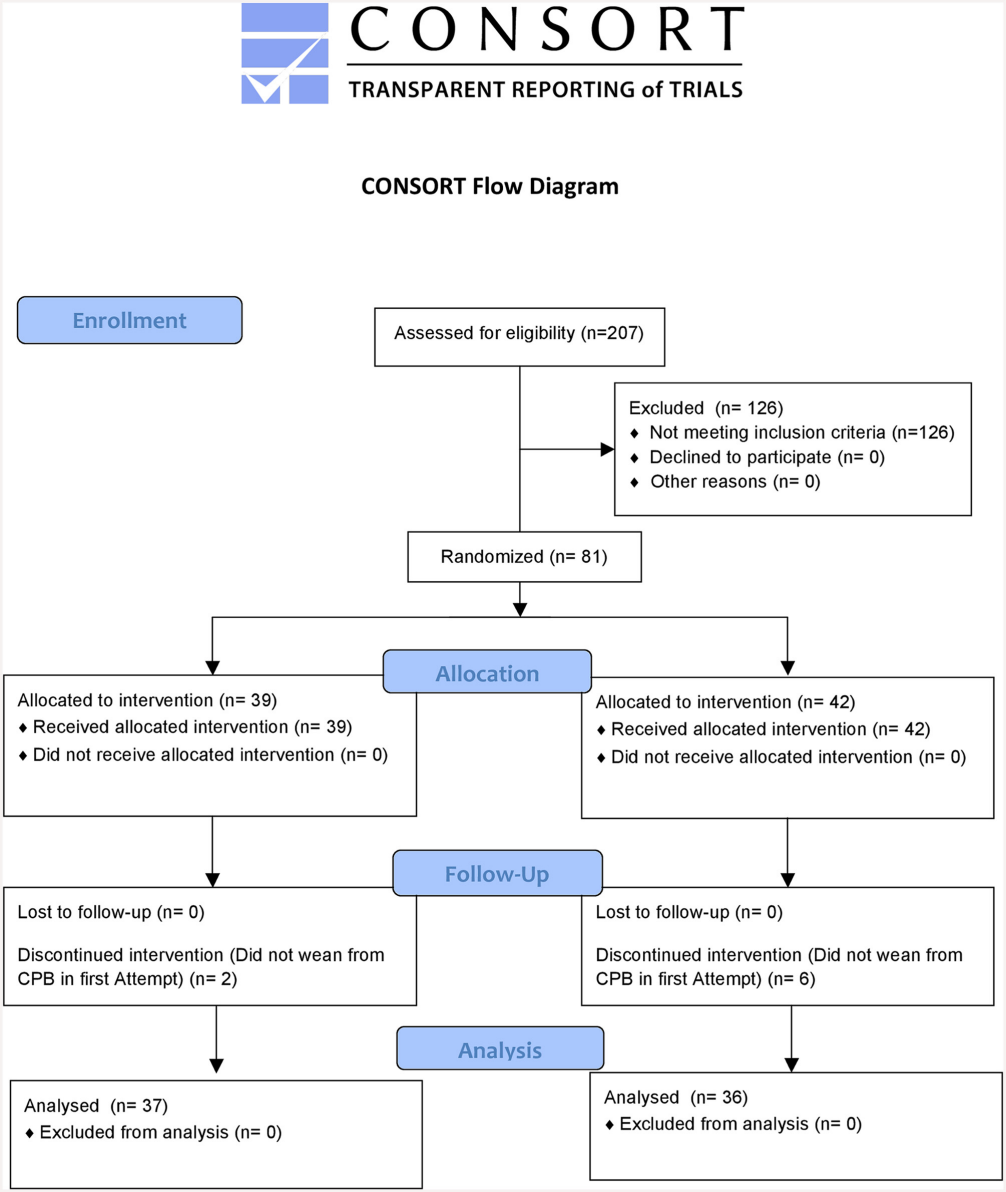
CONSORT diagram. CBP: Cardiopulmonary by-pass.

This prospective, randomized and double-blind clinical trial was conducted at the National Cardiology Institute of Brazil. This study was released by the Ethics Committee of the National Cardiology Institute following the declaration of Helsinki and was registered with Clinical Trials/FDA (www.clinicaltrials.gov; Identifier: NCT01616069). All participants signed an Informed Consent Form and the author states that the report includes every item in the CONSORT diagram and checklist for a prospective randomized clinical trial (Fig 1).

After enrollment in the research, eighty-one patients who underwent on-pump CABG surgery between January 2011 and July 2013 were computer-generated using a randomization allocation rate 1:1, divided into 2 groups: epinephrine group and levosimendan group, to evaluate left ventricle MPI. The randomization list was computer-generated by the GraphPad Randomize1^®^ (GraphPad Software^®^, Inc., La Jolla, CA, USA). The researcher and the echocardiographer were blinded to the protocol of the inotropic drug that was used. They performed only the echocardiographic exams.

Another anesthesiologist was responsible for anesthetic procedures, including the preparation and infusion of the solution containing the drug studied, according to the list generated by the electronic randomization. These anesthesiologists did not collect or interpret any data in this study.

The patients were of both genders, aged between 40 and 65 years, and had a heart rate (HR) between 50 and 90 beats per minute (sinus rate) when using beta blockers. The patients had an ejection fraction (EF) > 35% and a hematocrit level > 30%.

The exclusion criteria during the preoperative period were as follows: moderate or serious valve disease; malignant neoplasm; glycemia > 200 mg/dl, creatinine > 2.0 mg/dl, or bilirubin > 3.0 mg/dl; and patients undergoing cardiac reoperation surgeries or CABG associated with valve and carotid surgeries. Patients who were not weaned from CPB at the first attempt were also excluded from the study. There was no interruption in any routine-use medication for the patients (except for clopidogrel, which was suspended for at least 5 days before surgery). The preoperative EuroSCORE and data regarding related diseases, such as systemic arterial hypertension, smoking, diabetes mellitus, previous acute myocardial infarction, and preoperative angioplasty, were recorded.

### Anesthetic and echocardiographic technique

Monitoring was performed using electrocardiogram, pulse oximeter, and capnograph. Invasive blood pressure, central venous pressure, bispectral index and central temperature were determined. After monitoring, all patients received general anesthesia with etomidate, cisatracurium, and fentanyl. Tracheal intubation was performed and 8-MHz multiplane intraoperative transesophageal echocardiogram probe (Vivi I^®^, GE, Helsinki, Finland, 2011) was introduced, and the echocardiographic study began, following the protocols of the Society of Cardiovascular Anesthesiologists [21, 22] and American Society of Echocardiography [23]. All exams were performed by the researcher and analyzed by a single echocardiographer. The researcher and echocardiographer were blinded to the study. Sevoflurane, cisatracurium and continuous infusion of fentanyl were used for maintenance of anesthesia. During the CPB, sevoflurane was replaced for a continuous infusion of propofol.

**Fig 2:**
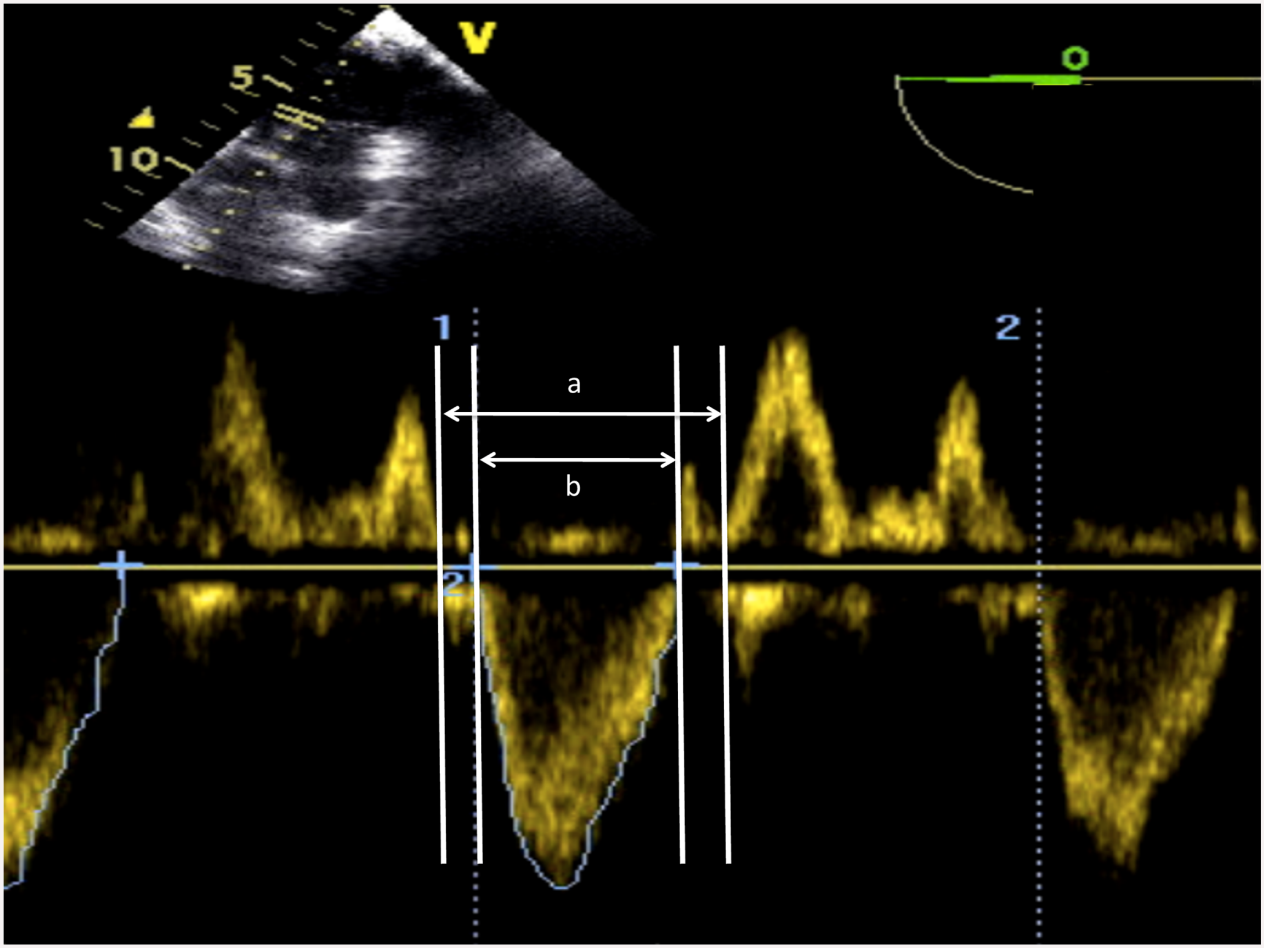
Schematic representation of the measurement of the left ventricle myocardial performance index. a = distance between the mitral A wave and E wave; b = distance between the start and end of the flow of the left ventricle outlet tract (LVOT).

The left ventricle myocardial performance index was calculated by pulsed Doppler imaging between the anterior leaflet of the mitral valve and the left ventricle outflow tract (LVOT) in the deep transgastric view: left ventricle myocardial performance index = (a-b)/b (a = distance between the mitral A wave and E wave; b = distance between the start and end of the LVOT flow (Fig 2) [12, 23].

Assuming that the LVOT is circular in shape, the LVOT cross-sectional area was measured in the middle esophagus long-axis view. The stroke volume and cardiac output were determined using the continuity equation in LVOT at each time point of the data collection [23]. The velocity time integral of LVOT was determined by pulsed Doppler imaging in the deep transgastric view [23]. The stroke volume was calculated by the equation: stroke volume (cm^3^) = cross-sectional area (cm^2^) x velocity time integral (cm). The cardiac index was determined by the equation: cardiac index = (stroke volume x heart rate)/m^2^ [23].

The ejection fraction was determined in the middle esophagus in the four and two-chamber views by measuring the left ventricular end-diastolic volume and the left ventricular end systolic volume by Simpson's method: ejection fraction = [(left ventricular end-diastolic volume − left ventricular end systolic volume) ÷ left ventricular end-diastolic volume] x 100%. The E/A ratio was determined by pulsed Doppler imaging of the mitral annulus through the middle esophagus four-chamber view. The e' value was calculated by tissue Doppler imaging of the lateral mitral annulus in the middle esophagus four-chamber view [23].

The following parameters were evaluated 30 minutes after tracheal intubation before chest opening (pre-CPB period), and 10 minutes after the end of protamine infusion after CPB (post-CPB period): left ventricle myocardial performance index, stroke volume, cardiac index, systemic vascular resistance index, left ventricular ejection fraction, E/A and E/e’ [24]. At these time points, we also noted the mean arterial pressure, central venous pressure, and heart rate.

All measurements were performed 3 times and was used the average of them. During the measurements, the patient had normal sinus cardiac rhythm, without atrium-ventricular pacing and was disconnected from mechanical ventilation [24, 25].

### Preparation of drugs

The researcher and the echocardiographer were blinded to the protocol of the inotropic drug that was used and they performed only the echocardiographic exams. After induction of anesthesia, other anesthesiologists, who did not collect or interpret any data of the study was responsible for the patient’s allocation to the epinephrine group or levosimendan group, according with the electronic randomization described above.

The solutions containing the drugs studied were prepared by the anesthesiologist, who performed the anesthetic technique using a 0.9% saline solution at a final volume of 100 ml. This solution was infused by an injection pump (Abbott, Sao Paulo, Brazil) using opaque (photosensitive equipment, because the color of levosimendan), and the infusion rate was the same in both groups (10 ml.h^-1^.). There was no loading dose of levosimendan or epinephrine [7, 26]. Infusion began when the patient reached 33°C during the rewarming [26]. In this study were used the previously published optimal doses of epinephrine (Adren^®^, Hipolabor, São Paulo, Brazil, 2008) 0.06 mcg.kg^-1^.min^-1^ [24, 27, 28] and levosimendan (Sindax^®^, Abbott, São Paulo, Brazil), 0.2 mcg.kg^-1^.min^-1^ [26, 29–31].

### CPB intervention

During the CPB, the dosage of heparin was 4 mg.kg^-1^, and the activated clotting time was maintained > 480 seconds. The patients were cooled to 32°C, and the CPB flow was calculated using the ideal cardiac index, which ranged from 2.0 to 2.5 L.min^-1^.m^2^. The myocardial protection was 4:1 blood cardioplegia in St. Thomas’^®^ solution at 4°C, injected into the aortic root every 20 min in all patients. During the CPB period, mean arterial pressure between 50 and 80 mmHg, urinary output > 1 ml.kg^-1^.min^-1^, hemoglobin > 7 g.dL^-1^, and venous return saturation > 70%. Arterial hypertension was treated with sodium nitroprusside, and arterial hypotension was treated with norepinephrine.

All patients were weaned from CPB using only epinephrine or levosimendan following randomization. The goals adopted to guide the separation from CPB included the patient being rewarmed until 36.5°C, normal sinus cardiac rhythms, cardiac index > 2.2 L.M^2-1^.min^-1^ and central venous pressure below 12 mmHg [3]. After total separation from CPB and removal of the aortic cannula, protamine was infused at a ratio of 1 mg per 100 IU of heparin.

The CPB volume reposition was controlled by the central venous pressure until 12 mmHg and the E/e’ below 15.

If the patients in the epinephrine or levosimendan group were not weaned from CPB at the first attempt, they were excluded from the study and were administered another inotropic agent (dobutamine or milrinone) to improve myocardial function.

If the patients continued to exhibit hypotension (MAP < 60 mmHg) with preserved ventricular function after volume infusion, norepinephrine (0.1 mcg.kg^-1^.min^-1^) was used. For arterial hypertension (MAP > 100 mmHg), nitroglycerin (0.5 mcg.kg^-1^.min^-1^) was used. In these cases, the patients were not excluded from the study.

### Analysis of primary and secondary outcomes

The primary outcome was the left ventricle myocardial performance index in patients undergoing on-pump CABG surgery and treated with epinephrine or levosimendan. The secondary outcomes were mean arterial pressure, heart rate, central venous pressure, cardiac index, systemic vascular resistance index, left ventricle ejection fraction and diastolic dysfunction, incidence of post-operative acute myocardial infarction, stroke and acute kidney injury [32,48].

To analyze the MB-CK enzyme and troponin I concentrations by the fluorescent immunoassay method (Triage^®^, Alegre, San Diego, USA), blood was collected at the following times [29, 32, 33]: 30 minutes after tracheal intubation before chest opening, during the pre-CPB period, at the end of surgery after chest closure, and 24 hours after the end of surgery.

### Statistical analysis, sample size, and power of the study

The primary endpoint of this study was the left ventricular myocardial performance index (MPI) evaluation in the pre-CPB and post-CPB period [34]. Data were converted into mean ± SD for epinephrine (37 patients) and levosimendan group (36 patients), a total of 73 patients. The Student t- test for normally distributed unpaired samples was used to compare the two groups. The Graph-Pad Prism software, version 4.03 (GraphPad Software^®^, Inc., La Jolla, CA, USA) allowed the analysis [34].

Nominal values were transformed into medians and interquartile range and the proportion test for two samples (Instat Plus program^®^, version 3.036 for Windows) was used to analyze the gender, arterial hypertension, diabetes mellitus, smokers, acute myocardial infarction, norepinephrine requirement and weaning from CPB. The analysis of gender with and without complication after CABG was calculated with the diagnostic test from MedCalc Software 2015, version 15.8 –(Last modified: August 14, 2015). An 80% power, a 0.05 Type-I error and an allocation relation equal to one were admitted and provided for the calculation of the sample size for the two independent samples. Based on the size effect, estimated sample size, and type-I error, the Type-II error also was calculated. A two-tail P<0.05 was considered significant for all statistic tests.

## Results

Eighty one patients were recruted in the study. Eigth patients were excluded: 6 of them in the levosimendan group (5 patients did not achieve good left ventricle performance and needed additional dobutamine to improve the myocardial function, and 1 patient showed an atrium-ventricular blockage requiring atrium-ventricular pacing) and 2 patients in the epinephrine group (1 patient showed poor left ventricle performance and needed additional milrinone and an intra-aortic balloon, and 1 patient was weaned from CPB due to atrial fibrillation and maintained arrhythmia until the end of the surgery). Thus, 73 patients were included in the present study (37 in the epinephrine group and 36 in the levosimendan group).

The Table 1 shows the analysis of myocardial performance index type II error and confirms the post-CPB period significance between epinephrine and levosimendan (Type II error = 0.09), and the significance of epinephrine group between the pre and post-CPB period (Type II error = 0.0008).

**Table 1:** Epinephrine and levosimendan sample size and a type II error. MPI: Myocardial performance index; CPB: Cardiopulmonary by-pass.

Data	Variable analysis	Sample size	Cohen’s effect size	Type II error
Pre-CPB MPI	Epinephrine x Levosimedan	10132	0.05	0.95
Pos-CPB MPI	Epinephrine x Levosimedan	54	0.78	0.09
Epinephrine MPI	Pre-CPB x Post-CPB	24	1.22	0.0008
Levosimendan MPI	Pre-CPB x Post-CPB	392	0.28	0.78

A significant lower left ventricle MPI (P = 0.0001) was observed in the epinephrine group between pre-CPB and pos-CPB period (Table 2).

**Table 2:** Epinephrine and levosimendan group mean and standard deviations in each moment (pre and post-CPB). CPB: Cardiopulmonary by-pass, MPI: myocardial performance index.

Data	Group	Pre-CPB	Post-CPB	p-Value
Left ventricle MPI	Epinephrine (n = 37)	0.43 ± 0.12	0.26 ± 0.15*	0.0001
Left ventricle MPI	Levosimendan(n = 36)	0.44 ± 0.19	0.39 ± 0.17	0.23

Table 3 shows a lower left ventricle myocardial performance index in epinephrine group in the post-CPB period when compared to levosimendan group (P = 0.0013).

**Table 3:** Myocardial performance index between epinephrine and levosimendan in the pre and post-CPB. CPB: Cardiopulmonary by-pass, MPI: myocardial performance index, NS: Not significant.

Data	Period	Epinephrine (n = 37)	Levosimendan (n = 36)	p-Value
Left ventricle MPI	Pre-CPB	0.43 ± 0.12	0.44 ± 0.19	NS
Left ventricle MPI	Post-CPB	0.26 ± 0.15*	0.39 ± 0.17	0.0013

Table 4 shows no significant difference between the groups in terms of patient characteristics and risk factors.

**Table 4:** Patient characteristics and risk factors. LVEF = left ventricle ejection fraction, SAH = systemic arterial hypertension, AMI = acute myocardial infarction. p > 0.05 for all variables analyzed.

Data	Epinephrine	Levosimendan
Total number of patients, n	37	36
Age, y	58 ± 5.6	58 ± 5.3
Body area, M^2^	1.8 ± 0.1	1.8 ± 0.1
Male, n (%)	33 (89)	29 (80)
Female, n(%)	4 (11)	7 (20)
Weight, kg	77 ± 9.5	76 ± 9.5
Height, cm	167 ± 8	167 ± 8.4
LVEF, %	50 ± 7.1	53 ± 9.2
Obstructed coronaries, n	3.6 ± 0.6	3.5 ± 0.8
EuroSCORE	2.0 ± 1.9	1.7 ± 1.3
SAH, n (%)	34 (91)	35 (97)
Diabetes mellitus, n (%)	18 (48)	16 (44)
Smokers, n (%)	17 (45)	19 (52)
Preoperative AMI, n (%)	19 (51)	23 (63)

When the hemodynamic data were analyzed in each group between pre-CPB and post-CPB periods (Table 5), the epinephrine group showed a higher heart rate, a higher central venous pressure, a higher stroke volume, a higher cardiac index, a lower systemic vascular resistance index and a higher ejection fraction in four and two-chamber view when were compared the pre-CPB period with post-CPB period. The levosimendan group showed a higher mean arterial pressure, a higher heart rate, a higher stroke volume, a higher cardiac index, a lower systemic vascular resistance index and a higher ejection fraction in a four-chamber view when were compared the pre-CPB period with post-CPB period.

**Table 5:** Hemodynamic data for the epinephrine and levosimendan group. MPI = myocardial performance index; HR = heart rate; MAP = mean arterial pressure; CVP = central venous pressure; SV = systolic volume; CI = cardiac index; SVRI = systemic vascular resistance index; LVEF, ME 4C, and ME 2C = left ventricle ejection fraction in the mid-esophagus, 4-chamber and 2-chamber views, respectively.

Data	Group	Pre-CPB	Post-CPB	p-Value
HR (bpm)	Epinephrine (n = 37)	60 ± 7.7	82 ± 12.8*	0.001
HR (bpm)	Levosimendan (n = 36)	57 ± 12.8	77 ± 10.4*	0.001
MAP (mmHg)	Epinephrine (n = 37)	68 ± 10.6	67 ± 9.5	0.47
MAP (mmHg)	Levosimendan (n = 36)	73 ± 10.8	68 ± 6.0*	0.023
CVP (mmHg)	Epinephrine (n = 37)	7 ± 4.7	10 ± 3.7*	0.004
CVP (mmHg)	Levosimendan (n = 36)	9 ± 8.8	10 ± 4.3	0.18
SV (ml/beat)	Epinephrine (n = 37)	52 ± 11.6	69 ± 13.5*	0.0001
SV (ml/beat)	Levosimendan (n = 36)	54 ± 16.6	64 ± 17.8*	0.013
CI (L.min^-1^.m^2^)	Epinephrine (n = 37)	1.7 ± 0.4	3.0 ± 0.7*	0.0001
CI (L.min^-1^.m^2^)	Levosimendan (n = 36)	1.6 ± 0.5	2.6 ± 0.8*	0.0001
SVRI (dyn.s^-1^.cm ^5^.m^2^)	Epinephrine (n = 37)	3064 ± 897.8	1574 ± 471.3*	0.0001
SVRI (dyn.s^-1^.cm^-5^.m^2^)	Levosimendan (n = 36)	3328 ± 1046	1907 ± 644.9*	0.0001
LVEF ME 4C (%)	Epinephrine (n = 37)	52 ± 14.1	65 ± 10.4*	0.0001
LVEF ME 4C (%)	Levosimendan (n = 36)	54 ± 13.2	62 ± 13.4*	0.019
LVEF ME 2C (%)	Epinephrine (n = 37)	52 ± 13.0	65 ± 11.4*	0.0001
LVEF ME 2C (%)	Levosimendan (n = 36)	55 ± 13.5	59 ± 15.6	0.25

*p < 0.05 in the same group between pre-CPB and post-CPB period (paired and unpaired Student *t* test).

**Fig 3:**
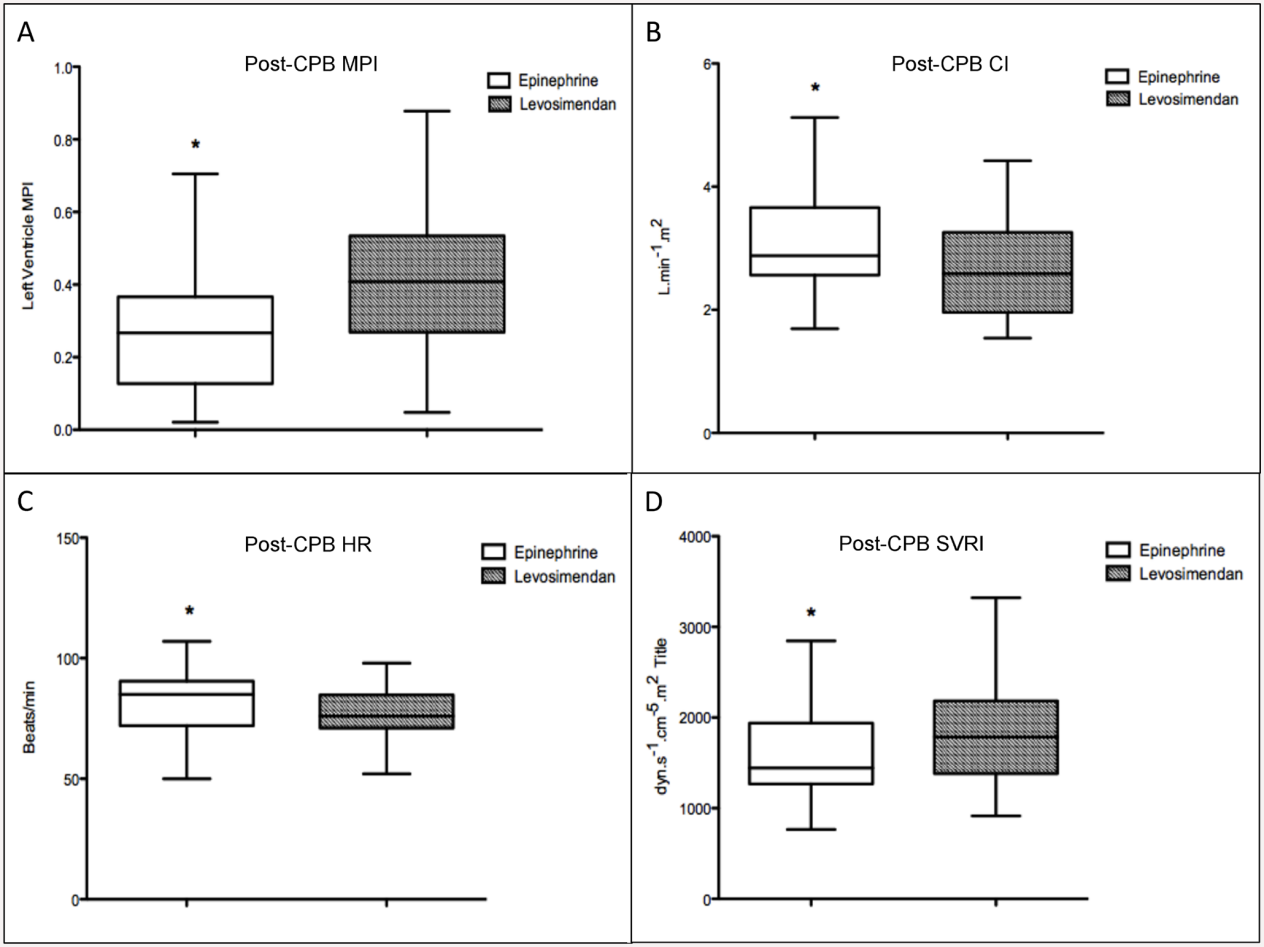
Assessment of epinephrine and levosimendan in the post-CPB period. **(A)** Assessment of the left ventricle myocardial performance index at the post-CPB period for the epinephrine group and levosimendan group. *p = 0.0013 (Student’s *t*-test) **(B)** Assessment of the cardiac index at the post-CPB for the epinephrine group and levosimendan group. *p = 0.03 (Student’s *t*-test) **(C)** Assessment of the heart rate at the post-CPB for the epinephrine group and levosimendan group. *p = 0.04 (Student’s *t*-test) **(D)** Assessment of the systemic vascular resistance index at post-CPB protamine for the epinephrine group and levosimendan group. *p = 0.01 (Student’s *t*-test). CBP: Cardiopulmonary by-pass, MPI: myocardial performance index CI: cardiac index, HR: heart rate, SVRI: systemic vascular resistance index.

Fig 3.A–3.D shows the hemodynamic data for the epinephrine group and levosimendan group during the post-CPB period. When the groups were compared, the epinephrine group had a lower left ventricle myocardial performance index (0.26 ± 0.15 vs. 0.39 ± 0.17; P = 0.0013), a higher cardiac index (3.06 ± 0.7 vs. 2.67 ± 0.8 L.min^-1^.m^2^; P = 0.03), a higher heart rate (82 ± 12.8 vs. 77 ± 10.4 bpm; P = 0.04) and a lower systemic vascular resistance index (1574 ± 471.3 vs. 1907 ± 644.9 dyn.s^-1^.cm^-5^.m^2^; P = 0.01) than levosimendan group during the post-CPB period.

Table 6 shows the diastolic and filling data for the left ventricle, and there was no significant difference between the epinephrine group and the levosimendan group.

**Table 6:** Diastolic and filling data for the left ventricle for the epinephrine and levosimendan group. Lateral e’ = lateral mitral valve annulus tissue Doppler velocity; E/e’ = relationship between the mitral E wave and the lateral e’ mitral valve annulus tissue Doppler velocity; E/A = relationship between the E wave and A wave by pulsed Doppler imaging in the mitral ring; DT = deceleration time for the mitral E wave by pulsed Doppler imaging.

Data	Group	Pre-CPB	Post-CPB	p-Value
Lateral e’(cm/s)	Epinephrine (n = 37)	5.9 ± 1.7	7.5 ± 3.0*	0.004
Lateral e’(cm/s)	Levosimendan (n = 36)	5.7 ± 2.0	6.5 ± 2.0	0.05
E/e’	Epinephrine (n = 37)	10.4 ± 2.9	12.6 ± 4.0*	0.0096
E/e’	Levosimendan (n = 36)	11.3 ± 4.0	13.1 ± 6.0	0.16
E/A	Epinephrine (n = 37)	1.2 ± 0.3	1.4 ± 0.6	0.11
E/A	Levosimendan (n = 36)	1.3 ± 0.4	1.3 ± 0.5	0.63
DT (ms)	Epinephrine (n = 37)	218 ± 52.8	189 ± 64.4*	0.04
DT (ms)	Levosimendan (n = 36)	227 ± 51.4	191 ± 43.2*	0.001

*p < 0.05 in same group between pre-CPB and post-CPB period (paired and unpaired Student *t* test).

Table 7 shows the time of CPB, the time of aorta cross clamp and the number of coronary anastomoses. There were not significantly different between the two groups. The norepinephrine requeriment was higher in the levosimendan than epinephrine group (16% vs. 47%; p = 0.005) in post-CPB period.

**Table 7:** Evaluation of aortic cross clamp, CPB time and post-CPB norepinephrine requeriment for the epinephrine and levosimendan group. Post-operative MI = post-operative myocardial infarction; AKI = acute kidney injury, CPB time = period of cardiopulmonary bypass.

Data	Epinephrine (N = 37)	Levosimendan (N = 36)	p-Value
Post-operative MI, n (%)	7 (18)	4 (11)	0.51
AKI, n (%)	3 (8)	4 (11)	0.71
Stroke, n (%)	1 (2)	1 (2)	1
CPB time, min	89 ± 16.8	89 ± 21.5	1
Aorta cross clamp, min	76 ± 17.5	78 ± 20.6	0.9
Drug infusion time until weaning from CPB, min	37.9 ± 7.3	41.3 ± 8.3	0.21
Norepinephrine requeriment, n (%)	6 (16)	17 (47)	0.005*
Coronary anastomoses, n	3.5 ± 0.6	3.5 ± 0.9	0.9

* p< 0,05 (Paired and unpaired Student *t* test).

Twenty-four hours after surgery, the plasma levels of troponin I (epinephrine group: 4.5 ± 5.7 vs. levosimendan group: 2.5 ± 3.2 g/dl; p = 0.09) and MB-CK (epinephrine group: 50.7 ± 31 vs. levosimendan group: 37 ± 17.6 g/dl; p = 0.08) were not significantly different between the two groups.

The analysis of sensitivity for clinical outcomes in the postoperative period was done in the men group. A positive likelihood ratio (0.05) and a negative one (1.92) for the epinephrine treated men group provided no convincing evidence of sensitivity for the clinical outcomes. The levosimendan treated men group showed the same result with a positive like a positive likelihood ratio (0.17) and a negative on (1.76)

There was no in-hospital mortality.

## Discussion

The primary goal of this study was to evaluate the effects of epinephrine and levosimendan on left ventricle myocardial performance index in patients undergoing on-pump CABG surgery. Myocardial performance index or the Tei index, measured by Doppler echocardiography, consists of the sum of the isovolumetric contraction time and the isovolumetric relaxation time, divided by the ejection time [11, 12, 35]. Myocardial performance index has an important correlation with systolic and diastolic functions of the left ventricle, which can guide the use of inotropic drugs and loading conditions during the weaning from CPB [8–10]. Myocardial performance index analyzed by linear regression has an inverse correlation with the cardiac index and a direct correlation with the systemic vascular resistance index [10, 15]. Similar to other studies, patients who underwent CABG surgery and showed a left ventricle performance index of 0.47 or less had better clinical outcomes and lower mortality [14, 17]. In the present study, average values of the left ventricle performance index in epinephrine group was 0.26 ± 0.15 and levosimendan group was 0.39 ± 0.17, which could explain the higher incidence of weaning from CPB at first attempt. However, in the current study, the epinephrine group showed a significant reduction in the left ventricle myocardial performance index value during the post-CPB period (epinephrine group: 0.26 ± 0.15 vs levosimendan group: 0.39 ± 0.17; p = 0.0013), thus, a higher incidence of weaning from CPB in the first attempt (95% vs 85%, p = 0.0001) when compared to levosimendan group, although some studies demonstrated that levosimendan can improve the weaning from CPB when compared with placebo, milrinone and intra-aortic ballon [3, 31, 36].

The metabolite of levosimendan (OR-1896) can improve cardiac performance for up to a week. Some authors showed that levosimendan facilitated the weaning from CPB and its effect on cardiac performance index could continue during the intensive care period, until one week or more, lead a better outcomes and lower mortality in the postoperative period [3, 37, 38]. However, in the present study, the levosimendan and epinephrine were not accessed in the postoperative period.

During the weaning from CPB, cardiac index was higher in both groups [2, 24, 39]. However, in our study, it was noted a higher cardiac index in the epinephrine group than levosimendan group in the post-CPB period, as was showed by Ravikumar [2]. So, the higher cardiac index in the epinephrine group compared to levosimendan group are associated with a lower left ventricle myocardial performance index in the epinephrine group caused by increase in ejection time [3, 29, 31], resulting a better myocardial performance.

In this study, systemic vascular resistance index during the weaning from CPB was lower in both groups and epinephrine group showed a lower systemic vascular resistance index when compared to levosimendan group in the post-CPB period. Inodilation action of levosimendan can frequently lead to reduction in systemic vascular resistance by phosphodiesterase type III inhibition, open ATP-sensitive K^+^ channels and nitric oxide release [2, 26, 26, 40]. In the present study, even with optimal loading conditions, to avoid systemic arterial hypotension we used a continuous infusion of norepinephrine when mean arterial pressure remained below 60 mmHg; thus, levosimendan group required more norepinephrine than epinephrine group (epinephrine group: 16% vs. levosimendan group: 47%; p = 0.001) [2, 5, 40]. Therefore, increase in systemic vascular resistance index in levosimendan group compared with epinephrine group at post-CPB period might have been caused by the higher amount of norepinephrine administered in levosimendan group. The myocardial performance index analyzed by linear regression has a direct correlation with the systemic vascular resistance index [15, 41]. So, a higher systemic vascular resistance index is associated with a higher left ventricle myocardial performance index because of the increase in isovolumetric contraction time [3, 29, 31].

In the present study, diastolic values between the two groups were not different during the post-CPB period. Diastolic function can be impaired by the CPB with cardioplegic arrest which leads to myocardial edema [24]. Lusitropic effects of levosimendan and epinephrine [39] could improve the ventricular loading, but there were no difference in diastolic function in post-CPB period in both groups. This could be happened, because the data were collected in the post-CPB period. Myocardial edema may be present for 6 hours after CPB cardioplegic arrest [24], and as the data were collected immediately after CPB weaning, there were no time to improve left ventricle relaxation by epinephrine or levosimendan. Deceleration time of the mitral E wave was reduced in both groups during the post-CPB period, probably due to increase in heart rate and the pre-load conditions in both groups. Although the lateral e’ showed an increase in epinephrine group and a tendency to increase in levosimendan group [42], there was no difference in loading conditions in both groups, which were observed central venous pressure and E/e’ at the post-CPB period [43].

All patients exhibited normal cardiac sinus rhythms without atrium-ventricular pacing during the pre-CPB and post-CPB data collection. Both groups showed increase in heart rate during post-CPB period, but epinephrine group had a 5 beats/min higher rate than levosimendan group. (epinephrine group: 82 vs. levosimendan group: 77 beats/min; p = 0.001). This results is similar to others studies [2, 7].

According to Antila et al. [44] levosimendan has a rapid onset time (< 30 minutes). The BELIEF study [45] demonstrated no difference in inotropic action of levosimendan when it was used continuously or as a bolus. In this study, we used continuous infusion of both drugs without a loading dose. The data were collected during the post-CPB period, when the epinephrine infusion was 37 minutes and that of levosimendan was 41 minutes after the drugs infusion had begun.

Previous studies and meta-analyses [8, 19, 20, 46, 47] that compared levosimendan, dobutamine, milrinone, and placebo treatment concluded that patients who used levosimendan showed myocardial protection with lower incidence of myocardial infarction, lower post-operative release of troponin and lower in-hospital mortality; however, other studies did not demonstrate differences in clinical outcomes from treatment with levosimendan or other inotropic drugs [2,10, 33].

Although some studies showing a higher incidence of clinical complications after CABG correlation with diabetes and female patients [48], in the present study, there were no significant differences in both groups regarding the clinical outcomes of the plasma levels of troponin I and the MB-CK 24 hours after surgery and there was no intra-hospital mortality.

### Limitations

This study has certain limitations. The data were collected in the pre- and post-CPB period, and there was no follow-up of the hemodynamic parameters in the postoperative period. These data are relevant because the metabolism of levosimendan (OR-1896) can lead to improve cardiac performance for up to a week. This study was performed on a population with left ventricle ejection fraction > 35%; therefore, the results cannot be applied to patients with severe left ventricle dysfunction.

### Conclusion

Although both drugs have improved the myocardial function and showed a safety profile to facilitate weaning from CPB, the epinephrine group showed a lower myocardial performance index, a higher cardiac index, a lower systemic vascular resistance index and a higher heart rate than levosimendan group. The epinephrine group also showed a higher incidence of weaning from CPB in the first attempt than the levosimendan group in the post-CPB period [10, 37].

## Supporting Information

S1 CONSORT Checklist CONSORT checklist. (DOC)

S1 Text Research Project. (DOC)

S1 Table Data table. Legend of data table. EF: Ejection Fraction; DM: Diabetes Mellitus; SAH: Sistemic arterial hipertension; Pre-AMI: Pre-acute myocardial infarction; CPB: cardiopulmonary by-pass; Clamp: aorta clamp; Use Norep: norepinephrine requeriment; T0: Pre-CPB; T1: Post-CPB; HR: heart rate; MAP: mean arterial pressure; CVP: central venus pressure; SV: stroke volume; CI: cardiac index; SVRI: sistemic vascular resistence index; EF 4C: ejection fraction 4 chamber; EF 2C: ejection fraction 2 chamber; E/A: relation mitral E and A wave; DT E: desaceleration time of mitral E wave; E’: Tissure doppler of lateral mitral annulus; E/e’: relation between mitral E wave and e’; MPI LV: myocardial performance index of left ventricle; HV: cristaloid infusion; Tropo: Troponin I value; MB: creatino kinase MB fraction; ICU: Intensive care unit; AMI: acute myocardial infarction; AKI: acute kidney injury.
(XLS)
