# Supplementary material for: A Randomized Blinded Study of the Left Ventricular Myocardial Performance Index Comparing Epinephrine to Levosimendan following Cardiopulmonary Bypass
Source: PLoS One. 2015 Dec 14;10(12):e0143315. doi: 10.1371/journal.pone.0143315 (PMC4684363; doi:10.1371/journal.pone.0143315)
Supplement: S1 Text — (DOC) [file pone.0143315.s002.doc]

**RESEARCH PROJECT**

**Assessment of adrenaline and levosimendan using transesophageal echocardiography in on-pump CABG Surgery**

Researchers: Professor Nubia Verçosa Figueiredo

                          Professor Ismar Lima Cavalcante

                          Dr. Marcello Fonseca Salgado Filho

Federal University of Rio de Janeiro

Department of Surgery

Rio de Janeiro

October 2011

**Assessment of adrenaline and levosimendan using transesophageal echocardiography in on-pump CABG Surgery**

Introduction

The first association between coronary heart disease and myocardial dysfunction was suggested by Parry in 1799, which described the death associated with angina and the presence of "ossification" of obstructive coronary arteries in his autopsy findings. Other authors, such as Heberden and Virchow in 1802, also describe the association of chest pain associated with WORKOUT and the presence of fibrous thickening of the walls of the coronary arteries due to an inflammatory reaction, which triggers a local fibroblast proliferation with arterial obstruction and evolves into a frame of acute myocardial infarction (AMI). Acute myocardial infarction is one of the diseases that most often progresses to heart failure.

The diagnosis of perioperative ventricular dysfunction occurs: when there is systolic blood pressure less than 90 mmHg; arteriovenous difference greater than 5,5ml / dL; cardiac index less than 2.2 L / min / m2, and pulmonary capillary wedge pressure greater than 15 mmHg.

The routine use of beta-blockers such as medical treatment, improves the prognosis of patients coronary artery disease. When associated with coronary artery bypass grafting (CABG) with cardiopulmonary bypass (CPB), triggers a decrease in the concentration of beta receptors (β) heart that aggravate ischemic ventricular dysfunction.

In the systematic review by Griffin and Hines, inotropic drugs and inodilators (dobutamine, epinephrine, milrinone and levosinmedan), and mechanical devices (intra-aortic balloon pump, artificial ventricle and extracorporeal membrane oxygenation -ECHMO) are able to improve the function of left ventricle in the perioperative period.

The inotropic beta-adrenergic such as adrenaline at lower doses and dobutamine, are world drugs used with good clinical evidence. The half-life short of these drugs, allows discontinuation or adjustment of intravenous infusion, when diagnosed any adverse effects, such as hypotension, hypertension and arrhythmias. These drugs improve cardiac function by cyclic adenosine monophosphate (cAMP) and consequent increase in intracellular calcium. Increased ventricular contraction force provides a greater myocardial oxygen consumption, lower arrhythmogenic threshold and reduction of myocardial protection. Any adrenaline, dobutamine as, at higher doses, platelet aggregation properties have, to a greater incidence of coronary thrombosis. Furthermore, dobutamine elicits action tachycardia β1 receptor in the sinoatrial node, which is more severe than that caused by adrenaline. The main consequence of the deleterious tachycardia is the largest myocardial oxygen consumption.

The inodilator, milrinone, is a derivative biperidino, phosphodiesterase type III inhibitory effects on: positive inotropic, increased ventricular diastolic time (lusinotropismo), systemic and pulmonary arterial vasodilation. Thus occurs: decrease in pulmonary capillary wedge pressure, improvement of cardiac index, increased venous oxygen saturation and blood flow of the coronary arteries. It also reduces the incidence of coronary spasm. It is one of the drugs recommended in cases of moderate to severe ventricular dysfunction, despite being associated with higher incidence of atrial fibrillation and hypotension.

The inodilator, levosimendan, the first generation calcium synthesizer cardiac troponin and agonist of ion channels triphosphate potassium-dependent adenosine (ATP-K +) is shown to increase myocardial contraction strength in patients with ventricular dysfunction for a period of up to 48 hours. Despite increase the strength of cardiac contraction, this drug does not increase myocardial oxygen consumption, and stable ventricular diastolic time. Due to stimulation of ATP-dependent K + channels occurs pulmonary arterial vasodilation and coronary arteries, thereby establishing a cardioprotective effect in patients with acute myocardial infarction undergoing coronary artery bypass surgery.

To assess the hemodynamic effects of drugs for treating myocardial dysfunction after CPB, the pulmonary artery catheter is used (CAP - Swan-Ganz), considered the gold standard monitoring. However, it has complications such as hematoma at the site of puncture, pneumothorax, pericardial effusion, heart valves and rupture sepsis, among others. In some clinical trials, patients monitored with the CAP had higher mortality than those who did not undergo such monitoring. In order to minimize these complications have been new technical proposals, considered less invasive. It stands out among these, transesophageal echocardiography (TEE).

The TEE is a hemodynamic diagnostic method with reduced incidence of complications when compared to the pulmonary artery catheter. The TEE is considered a monitoring which improves the clinical course of patients undergoing cardiac surgery. So it should be routinely used. Rate qualitatively and quantitatively the function of the ventricles of the heart valves, with the same accuracy PAC.

The TEE is a probe through the esophagus of the patient under sedation or general anesthesia. When the alternating voltage electric current passes through the quartz crystal, located in the distal portion of the probe generates a vibration in these crystals, called piezoelectric effect. Thus, it produces the ultrasonic waves that are emitted by the TEE probe and absorbed by the heart. These waves return to the probe, and at this time, there is the transformation of the ultrasonic wave received in two-dimensional image (2D).

ETE Doppler mode measures the blood flow velocity of the valve areas. Trans-pressure valve is calculated using the equation Bertoulli (P = 4V2). In this way, are calculated hemodynamic parameters

Background and Objectives

This study aims to compare through the ETE, the effects of adrenaline and levosimendan in patients with coronary obstruction, will be undergoing coronary artery bypass grafting with cardiopulmonary bypass, pre- and post extracorporeal circulation (ECC ).

The rationale of this study is the ability to choose the best drug to be used to optimize cardiac function in CPB in patients with moderate to severe ventricular dysfunction undergoing coronary artery bypass grafting using an ETE unit for measuring data hemodynamic, because this way we can improve the clinical outcome of the patient defining the best inotropic drug using a less invasive procedure and has lower mortality for measuring hemodynamic data.

Methods

 The study is the clinical, prospective, randomized double-blind which will be evaluated 60 patients of both genders, aged 40 to 65 years, undergoing coronary artery bypass grafting (CABG) with cardiopulmonary bypass (CPB) . The surgical indication for CABG with CPB obey the criteria of the Heart Surgery Service of the National Institute of Cardiology / Ministry of Health (INC / MS), located at Rua das Laranjeiras, 3000 in Orange, Rio de Janeiro, after clinical analysis and of laboratorial examinations (complete blood count, complete coagulation, plasma sodium, plasma potassium, urea and serum creatinine, blood glucose), electrocardiography at rest, chest radiography, echocardiography at rest and cardiac catheterization. All patients who agree to participate in the study will sign a Consent and Informed (IC) in the pre-anesthetic evaluation (Appendix 1).

Patients will be randomly divided into four groups computed medium with 40 patients:

GA: adrenaline,

GL: levosimendan.

All survey data will be noted in the protocol form (Appendix 2)

Patients are anesthetized by Dr. Marcello Fonseca Salgado Filho researcher who is also responsible for conducting the examination with the TEE. A random selection of groups and the preparation of drugs are made by other anesthesiologists INC / MS, by lot.

The pre-anesthetic evaluation will be made by the researcher, in two stages: in the clinic and on the eve of the anesthetic-surgical procedure. Patients will not receive premedication.

 In the operating room will be carried out sequentially, the following:

1. Non-invasive monitoring: cardioscope with two leads (D2 and V5), ST segment analysis, noninvasive blood pressure (NIBP), pulse oximetry, capnography with gas analyzer, bisespectral index (BIS) (Datex-Ohmeda® S / 5 Anesthesia Machine Aespire; Helsinki, Finland, 2009).

2. peripheral venoclysis: right or left arm with 14G catheter and started intravenous infusion of Ringer's lactate. Immediately after venipuncture, is injected intravenous midazolam (0.05mg / kg).

3. monitoring of invasive blood pressure (PAI): the radial artery compression and ulnar will be held simultaneously, and evaluated the absence of peripheral perfusion analyzed hand. Releases the compression of the ulnar artery and is observed, the return of peripheral perfusion (positive Allen test), proceed then the radial artery puncture with specific material (arterial® Cook-Kit, Germany, 2008) followed by the passage of guide wire, with subsequent introduction of the catheter 20 G which will be introduced in the radial artery (Seldinger technique). After the puncture, blood line is connected to the monitor (Datex-Ohmeda® S / 5 Anesthesia Machine Aespire; Helsinki, Finland, 2009) for measuring the FATHER.

4. monitoring of central venous pressure (CVP): the technique of choice will be the puncture of the right internal jugular vein using the Seldinger technique, with double lumen catheter 18 and 20G (Cook-Kit venipuncture Central®, Germany, 2008). After the puncture, the 20G venous line will be connected to the monitor (Datex-Ohmeda® S / 5 Anesthesia Machine Aespire; Helsinki, Finland, 2009) for measuring PVC.

5. Anesthesia: pre-oxygenation under facial mask, breathing spontaneously for five minutes; etomidate intravenous injection (0.3mg / kg), atracurium (0.2 mg / kg) and fentanyl (7mcg / kg). Next, the manual ventilation will be held a face mask for 5 minutes, and tracheal intubation. Patients will be mechanically ventilated by anesthesia machine (Datex-Ohmeda® S / 5 Anesthesia Machine Aespire; Helsinki, Finland, 2009), with controlled ventilation pressure of 20 cm H2O, respiratory rate of 12 breaths per minute, fraction of inspired oxygen 50 % and positive end-expiratory pressure (PEEP) of 3 mmHg.

6. maintenance of anesthesia: sevoflurane will be between 2% and 4% expired fraction, fentanyl (3-5 mcg / kg / h) and cisatracurium (2mcg / kg / min).

7. introduction of TEE: with the patient intubated, the oropharynx of patients will be lubricated with own jam (Savage Laboratories®, New York, USA) for the passage of the TEE probe (Vivi I®, GE, Helsinki, Phinland, 2011) . Examination started by viewing images of 20 recommended by the Society of Cardiovascular Anesthesiologist (SCA).

8. hemodynamic measurements:

8.1. stroke volume (SV): initially, will be needed to measure the sectional area (AS) and full speed by the time (VTI):

The calculation of VTI of the left ventricular outflow tract (LVOT) will be held by pulsed Doppler in the preview for trans-gastric cut deep. The diameter (D) of the LVOT is calculated in the long axis of the aortic valve by cutting in the middle the esophagus using the formula:

 AS (cm2) = 0.785 X D2.

Therefore, the stroke volume is calculated by the equation:

 SV (cm3) = S (cm2) x VTI (cm).

8.2. Cardiac output (CO) using the formula:

 DC = X VS FC.

8.3. Ejection fraction (EF): visualizing the cut of 4:02 heart chambers in the middle esophagus, will measure the left ventricular end-diastolic volume (LVEDV) and left ventricular end-systolic volume (LVFSV) and measured the FE the Simpson method (LVEDV-LVFSV / LVEDV).

8.4. E / A, E / e 'and TEI index: by pulsed Doppler through the mitral annulus and the tissue Doppler of the lateral wall of the left ventricle, near the posterior leaflet of the mitral valve, in the view of the four heart chambers in the medium esophagus .

9. inotropic solutions: following the previously drawn, the amine is prepared and infused by pump (Baxter Colleage 3®, Singapore, 2010), on the other anesthesiologist during the reheating of the patient, still on CPB.

All drugs are prepared in saline with 0.9% final volume of 100ml:

9.1. adrenaline solution: Two mL (2 mg) diluted in 98mL of saline solution at 0.9%; concentration of 20 mcg / ml; 0,05mcg / kg / min.

9.2. dobutamine solution: 40 ml (500mg) diluted in 60 ml of saline solution at 0.9%; concentration 5000mcg / ml; 10 mcg / kg / min

9.3. milrinone solution: 20 mL (20 mg) diluted in saline 80 mL of 0.9%; concentration of 200mcg / ml; 0,5mcg / kg / min

9.4. levosimendan solution: Two mL (100 mg) diluted in 98 ml of saline solution at 0.9%; concentration 100 mcg / mL; 0,2mcg / kg / min

10. extracorporeal circulation (ECC): The perfusionist will use the circuit and the membrane oxygenator (Braile Medical). The initial volume (priming) is 2000ml of Ringer's lactate solution. The CPB machine flow rate may vary from 2,5 to 3,5 ml / kg / m2. The patient remains at normothermia (36 ° C).

11. Collection of hemodynamic data: VS, FE, DC, IC, RVSI, E / A and E / e, Tei index, PAM, PVC and FC will be assessed at the following times:

11.1. T0: Thirty minutes after tracheal intubation,

11.2. T1: five minutes before cannulation of the aorta,

11.3. T2: Five minutes after protamine infusion,

11.4. T3: the end of surgery.

At the end of surgery, patients will be referred to the ICU intubated, keeping infusions of sympathomimetic amines in the following situations: CI <2.5 L / min / m2; MAP <80 mmHg; with epicardial temporary pacemaker and venous oxygen saturation <70%.

12. collection of biological markers for myocardial ischemia (Tropinina and cpk-mb):

12.1. M0: after invasive monitoring,

12.2. M1: the end of surgery,

12.3. M2: Twenty-four hours after M0,

12.4. M3: forty eight hours after M0.

The results up to five times higher than baseline (M0) will be considered as normal, exceeding this ratio will be considered postoperative myocardial ischemia.

13. validation of echocardiography: the examinations will be recorded on CD and then reviewed by a member of the Brazilian Society of Echocardiography covered for the study.

 Inclusion criteria of patients:

• aged 40 to 65 years.

• both genders

• coronary obstructive disease

• electrocardiogram showed sinus rhythm

• use of beta blocker

• Heart rate between 40 and 80 beats per minute before the anesthesia

• with ejection fraction greater than 35% and less than 50%

• with a higher hematocrit than 30g / dl

Exclusion criteria of patients:

• thyroid disease, diabetes mellitus

• malignancy in radiotherapy or chemotherapy

• blood levels of creatinine> 2.0 mg / dl and bilirubin> 3.0 mg / dl

• acute myocardial infarction in the month prior to surgery

• chest pain on admission to the operating room (emergency surgery)

• re-operations of cardiac surgery

• CABG surgeries associated with surgical aortic valve replacement and / or mitral, surgery on the carotid artery and / or aorta.

• BMI> 30

Statistical analysis

The parametric data will be analyzed by Student's t-test. Data nonparametric Mann-Whitney test and categorical data the chi-square test.

The program will be used SPSS version 16 for Windows. The statistical significance of p <0.05.

Results are presented as mean ± standard deviation

Sample size

The calculation for 30 patients per gupo matches (total 60 patients) to a confidence value of 6 to 95% probability.

Answering adverse events

Will be provided free and appropriate treatment for all patients included in the study that are experiencing adverse events (expected or not) resulting from the procedure performed in accordance with the approved study protocol.

Infrastructure and facilities

The National Institute of Cardiology is a specific body of the Ministry of Health. As Decree. 6860 27/05/2009 is an integral unit of the Health Care Secretariat, bound, technically and administratively to the State Health Minister, serving as Reference Center of High Complexity Cardiovascular the Ministry in accordance with Ordinance 210-15 / 06/2004. Are your responsibilities:

I - To participate in the formulation of the national policy of prevention, diagnosis and treatment of cardiac diseases;

II - Plan, coordinate and guide plans, projects and programs at national level compatible with the execution of prevention activities, diagnosis and treatment of cardiac diseases;

III - To develop and guide the implementation of training activities, training and improvement of human resources at all levels in cardiology;

IV - To coordinate programs and conduct clinical, epidemiological and experimental research in cardiology, cardiac surgery and the like;

V - Advise and provide assistance medical services in the area of ​​cardiology and the like;

VI - Establish technical standards for standardization, control and rationalization of the procedures adopted in the specialty.

VII - To encourage studies and promote research aimed at stimulating the expansion of knowledge and scientific production in cardiology, cardiac surgery and the like.

In addition, the INC is an integral unit of the National Center for High Complexity Adjustment (CNRAC) that caters to states that do not have services in sufficient quantity, or those which do not exist, to ensure people's access to another residence state.

The INC is now the only public hospital in the metropolitan area of ​​Rio de Janeiro which operates on a regular, systematic, quantitative and qualitatively in highly complex cardiovascular, one of Brazil's greatest centers performing congenital heart disease surgery.

To perform its duties, the INC set as its Mission and Objectives:

Mission: "To promote cardiovascular health, training professionals, develop and disseminate knowledge and technology for social and economic development of the country."

Objective: To provide high complexity procedures in the area of ​​cardiovascular disease, with equity of access, problem solving and generating quality assurance and disseminating knowledge through research, training and development of human resources.

The National Institute of Cardiology is the referral center for high complexity cardiovascular, providing adult and pediatric cardiovascular surgery, interventional cardiology and electrophysiology laboratory.

Effective staff of the INC, among other specialties, consists of:

• Board 2

• Board 10

• Doctors 455

• Nurses 219

• Nutritionist 18

• Physiotherapists 28

• Nursing Technicians 84

• Nursing Assistant 364

• Administrative 224

• Residents 41

• Social Worker 17

• Administrator 14

• Occupational Therapist 05

Now recognized throughout the medical profession as a referral hospital, the INC maintains the following services following listed below:

PROFILE HOSPITAL

• Adult Cardiac Surgery Service

• Children's Heart Surgery Service

• Anesthesia Service

• Care Unit Cardio-Surgical Intensive

• Cardio-Intensive Unit Clinic

• Postoperative Children Unit

• Coronary Care Unit

• Semi-intensive Unit

• Coronary Disease Service

• Cardiomyopathy Service

• Heart Failure and Transplant Service

• valvular disease Service

• Hypertension Service

• Arrhythmia Service

• Cardiology Services for Children and Adolescents

• CCIH

• Radiology Service

• Hemodynamics and Interventional Cardiology Service

• Echocardiography Service

• Nuclear Medicine Service

• Laboratory Procedures Service

• Hemonúcleo

• Ergometry Service

• Pathology Service

• Nutrition Service

• Pharmacy Services

• Social Service

• Dental Services

• Physiotherapy Service

• referees

• Nursing

• Rehabilitation

SPECIALTIES

Adult Cardiovascular Surgery

Pediatric Cardiovascular Surgery

Interventional Cardiology

Electrophysiology Laboratory

Heart Transplant

Complexity HIGH

• Clinical Analysis Laboratory

• Conventional Radiology

• Computed tomography - 64 slices

• Ultrasound for inpatients

• Echocardiography (adult and pediatric), 1 three-dimensional device

• Hemodialysis

• Molecular Biology Laboratory

• Cell therapy Laboratory

• Interventional Cardiology (adult and pediatric)

• Ergospirometry

• adult and pediatric cardiac surgery

• Map

• Holter

• Nuclear Medicine

• Electrophysiology Laboratory

• Rehabilitation Laboratory

THERAPEUTIC UNITS - BEDS

Pediatric ward 22

Adult ward - heart failure and transplantation 07

Adult ward - cardiomyopathy 08

Adult ward - Hemodynamic 04

Adult ward valvular 26

Adult ward - Arrhythmia 03

Adult ward - coronary artery disease 29

ICU child Postoperative 05

Neonatal intensive care unit 05

Children's intensive care unit 01

Intensive care / isolation 01

Intensive clinical cardiology unit 07

Coronary unit 12

Semi-intensive care unit (installation) 19

Adult postoperative 19

Isolation unit and adult transplant 01

Emergency service (under construction) 01

Total beds 172

MAIN LINES OF RESEARCH:

Endocarditis

Dilated Cardiomyopathy

Rheumatic Fever

Electrophysiology and Arrhythmia

Quality and Cardiac Surgery

Arterial Hypertension

Cell Therapies

New drugs in cardiology

Cardiovascular risk in adolescents

Outpatient services in 2010:

Outpatient medical appointments 74,562

Echocardiogram 16,689

Nuclear medicine examinations 2792

Stress test 1,662

Electrocardiogram 26,800

Holter 940

Map 1016

Radiology 14846

CT 2469

Clinical pathology 510,452

Hemodynamic

Catheterizations 3758

Angioplasty 701

Valvuplastias 68

Children 670

Electrophysiological study 146

Definitive pacemaker implant 145

Surgeries 1231

Adult 860

Children 371

Therefore, the National Institute of Cardiology responsible for compliance dictated by the resolutions of the National Health Council in conducting research with human beings.

Investigator Compensation

There will be no remuneration for the principal investigator and collaborators, as well as there will be no cost to the research subjects who participate voluntarily in the same.

Compliance with resolutions of the National Health Council

The principal investigator of the above referenced research acknowledges the content of the National Health Council Resolution 196/96 and its complementary rather on research with human beings and undertakes to comply with its procedures in full.

Responsibilities

The principal investigator will have obligations to the Ethics Committee in institutional research, compliance with the rules dictated by the resolutions of the National Health Council in the conduct of research involving humans, compliance with the terms of the Protocol and the required procedures for the fulfillment of Good Practices Clinics, observation of the rights of research subjects and full support to them.

Reimbursement forms

All selected research subjects and included in this research project will be reimbursed for all expenses associated with its participation such as transportation to conduct consultations and / or examinations and food.

Publication of results

The results of the research for this study will be published, whether favorable or not.

Budget

The development of the research will be based on the anesthetic / surgical procedure patients. The facilities, laboratories and equipment needed are made available to carry out the work in the institution setting of this study, and these routine services provided to patients with CABG with CPB indications. The prescribed medications as well as anest'sica technical and TEE, are already in regular use in the practice of CABG with CPB, thus there is no further obstacle to the patient or to the institution.

The following budget forecast will be presented, as is fitting to point out that not foreseen financing for the implementation of this research project, it will be conducted using the researcher.

| Discrimininition | Quantity | Unit value | Total value |
| --- | --- | --- | --- |
| Pen | 1 | R$ 2,00 | R$ 2,00 |
| pen drive | 1 | R$ 39,90 | R$ 39,90 |
| paper A4 (package) | 2 | R$ 10,00 | R$ 20,00 |
| Print ink | 1 | R$ 37,99 | R$ 37,99 |
| Binding | 4 | R$ 3,00 | R$ 12,00 |
| total: |  |  | R$ 111,89 |

Conflict of interest: No

SCHEDULE (MONTHS)

| Activities | 1 | 2 | 3 | 4 | 5 | 6 | 7 | 8 | 9 | 10 | 11 | 12 | 13 | 14 | 15 | 16 | 17 | 18 | 19 | 20 | 21 | 22 | 23 | 24 |
| --- | --- | --- | --- | --- | --- | --- | --- | --- | --- | --- | --- | --- | --- | --- | --- | --- | --- | --- | --- | --- | --- | --- | --- | --- |
| REV. Literature | X | X | X | X | X | X | X | X | X | X | X | X | X | X | X | X | X | X | X | X | X | X | X |  |
| Data colecting |  |  |  | X | X | X | X | X | X | X | X | X | X | X | X |  |  |  |  |  |  |  |  |  |
| Data Analysis |  |  |  |  |  |  |  |  |  |  |  |  |  | X | X | X | X |  |  |  |  |  |  |  |
| Results |  |  |  |  |  |  |  |  |  |  |  |  |  |  |  |  |  | X | X | X |  |  |  |  |
| Discussion |  |  |  |  |  |  |  |  |  |  |  |  |  |  |  |  |  |  |  |  |  |  |  |  |
| Publication |  |  |  |  |  |  |  |  |  |  |  |  |  |  |  |  |  |  |  | X | X | X | X | X |

**References**

Parry CH. An inquiry into symptoms and cause of the syncope angin commly called angina pectoris, illustrated by dissections. Bath: R. Cruttwell; London: Cadell and Davis, 1799

Herbeden W. Commentaries on the history and cure of disease. News-gate: London: T. Payne. 1802. Pectoris dolor. 70, 362-8

Schoen FJ, Padera RF Jr: Cardiac surgical pathology in Cohn, Edmunds Lh Jr, eds. Cardiac surgery in the adult. New York: McGraw-Hill, 2003:199-85.

Spencer FA, Meyer TE, Gore JM, Goldberg RJ. Heterogenicity in the management and outcomes of patients with acute myocardial infarction complicated by heart failure. Circulation 2002;105:2605–10.

Yusuf S, Theodoropoulos S, Dhalla N, Mathias C, Yacoub M. Effect of beta blockers on dynamic exercise in human heart transplant recipients. J Heart Transplant 1985;4(3):312–4.

Cleland JG. From left ventricular dysfunction to heart failure. Arch Mal Coeur Vaiss 1996;89(11):1397–402.

Vatner DE, Vatner SF, Nejima J, et al. Chronic norepinephrine elicits desensitization by uncoupling the beta-receptor. J Clin Invest 1989;84(6):1741–8.

McAlpine HM, Cobbe SM. Neuroendocrine changes in acute myocardial infarction. Am J Med 1988:61–6 11; 84(3A).

Griffin MJ, Hines RL. Management of Perioperative Ventricular Dysfunction. J Cardioth and Vascul Anesth, Vol 15, No 1 , 2001: pp 90-106

Clements FM, Harpole DH, QuillT, et al. Estimation of left ventricular volume and ejection fraction by two-dimensional transesophageal echocardiography: comparison of short axis imaging and simultaneous radionuclide angiography. Br J Anesth 1990;64:331

Savage RM, Lytle BW, Arason S et al. Introperative echocardiography is indicate in high-risk coronary artery by-pass grafting. Ann Thorac Surg. 1997;64:368-73

Shanewise JS, Cheung AT, Aranson S et al. ASE/SCA guidelines for performing a comprehensive intraoperative multiplane transesophageal echocardiography examination: recommendations of the American Society of Echocardiography Council for Intraoperative echocardiography and he Society of Cardiovascular Anesthesiologist Task Force for certification in perioperative transesophageal Ecocardiography. Anesth Analg, 1999;89(4):870-84

Urbanowicz JH, ShaabanMJ, Cohen NH, et al. Comparison of transesophageal echocardiographic and scintigraphic estimates of left ventricular end-diastolic volume index and ejection fraction in patients following coronary artery bypass grafting. Anesthesia, 1990; 72(4):607-12.

Salgado Filho MF, Miana LA, Siciliano A, Salgado J. O ecocardiograma transesofágico na cirurgia de Ross. Rev Bras Anestesiol, 2011; 100-101

APPENDIX 1

CONSENT AGREEMENT FREE AND SAVVY (196/96)

Comparative study of the hemodynamic effects of adrenaline, dobutamine, milrinone and levosimendan by transesophageal echocardiography in coronary artery bypass graft surgery.

Patient Name:

Chart:

Identity:

The purpose of this form is to obtain your consent to participate in research on a comparative study using medicines that improve heart function that will be analyzed by a device called echocardiography during heart bypass surgery.

This is a clinical trial testing a hypothesis that only have its defined benefit (if any) at the end of the study, after analyzing the data. There is no direct benefit to the participant. The draw to determine which group that Sr (a) will be part will be performed by a computer prior to surgery.

Routine procedures are:

• Clinical evaluation in the cardiac surgery service of the National Institute of Cardiology (INC).

• In the operating room will be held: puncture of a vein in the arm or hand; the radial artery in the wrist (blood pressure measurement and drawing blood for blood gas analysis). After use of a medicament for the Sr (a) drowsy, a vein is punctured in the neck for fluid infusion.

Drugs will be used to Sr (a) sleep and there will be the introduction of a tube in his throat to Mr (a) breathing aid apparatus (general anesthesia).

The surgery will be performed under general anesthesia with all comfort for pain and safety for you (a). After sleeping a probe through the mouth will be introduced until the stomach to assess the beating of his heart. This procedure will be done by an anesthesiologist (Dr. Marcelo F. Salgado Filho) of the National Institute of Cardiology (INC). During bypass surgery, it is necessary to make the heart of the Lord (a) stop beating, and your whole body will be controlled by a machine, so it can be possible to bypass. After performing this procedure, the heart begins to beat with the help of medicines.

During this surgery can occur arrhythmias (different beats) in the heart, major bleeding requiring transfusion do blood and allergic reaction to drugs to make you sleep. During the passage of the probe of the echocardiography device through the esophagus can occur damage to the esophageal wall with bleeding. The INC is properly prepared to address all these complications can happen by chance. At surgery, the Sr (a) will be sleeping with a tube into the trachea and breathing assistance devices (respirators), and referred to the Intensive Care Unit (ICU) under the supervision of surgeons and anesthesiologists.

At any stage of study, you will have access to responsible professional, Dr. Marcello Salgado Filho, which can be found at the National Institute of Cardiology on Mondays and Tuesdays. Contact phone (32) 99858833, INC: (21) -22853344

If you have any questions about the ethics of research please contact the Research Ethics Committee (CEP) / HUCFF / UFRJ, street Prof. Rodolpho Paulo Rocco, No. 255, University City, Fundão Island in time from 8 to 15 hours in room 01 D - 46 first floor, phone 25622480, email: cep@hucff.ufrj.br.

You participate voluntarily in this study, and will be free at any time, disagree, do not cooperate, give up participating in this survey or to withdraw your consent at any time, without prejudice to the continuity of their treatment in the institution.

To participate in this study you will not have personal expenses or to examinations or consultations. There will be also no payment for their participation in the study.

 In case of personal injury directly caused by the procedures or treatments proposed in this study, the participant is entitled to medical treatment in the institution as well as the legally established damages.

You can withdraw your consent or discontinue participation at any time. The researcher will treat your identity with professional standards of confidentiality and will not be identified in any publication that may result from this study.

The information obtained will be analyzed together with those of other patients. It is the researcher's competence in the evolution chart pledging to use the data and material collected only for this search.

The results of the study will be on hand when finalized and will be competence of the researchers involved in the project and professionals who may have service relationship and / or patient care and will not be allowed access to third parties, ensuring protection any type of discrimination or stigmatization.

 Your name or the material to indicate their participation will not be released without your permission guaranteeing the confidentiality of the information received or obtained in tests and observations by the researcher in relation to personal data of the research subject. Medical record data will also have privacy.

CONSENT:

I think I was sufficiently informed about the data on the aforementioned study I read or were read to me.

I discussed with Dr. Marcello about my decision to participate. Became clear to me what the purpose of the study, the procedures to be performed, their discomforts and risks, guarantees confidentiality and permanent clarification. It was also clear that my participation is free of cost and have guaranteed access to hospital treatment when necessary. Voluntarily agree to participate in this study and I can withdraw my consent at any time before or during the same, without penalty or loss and no loss of service in this institution or any benefit that I may have acquired. In addition, I am aware that I and the researcher in charge of this research we should initial all leaves this Term of Consent - IC.

Subject Search Name

                                                                                  Date / /

Signature of Subject Search

Researcher Name

                                                                                  Date / /

          The researcher's signature

APPENDIX 2

FILE protocol

NAME: ______________________________________________________________

GROUP: (____)

AGE: __________ YEARS

WEIGHT: _____________ KG

HEIGHT: ___________ CM

Body surface: ____________ M2

ASA: _______________

NYHA: ______________

CEC TIME: _______________ MIN

______________ MIN: DE CLAMP TIME

HT PRE: ___________ g / dl

HT POST: ____________ g / dl;

Blood products: CH (____); PLM (____); PLQT (____); CRIO (____)

| **datas** | **10 min after induction** | **10 min After Protamine** |
| --- | --- | --- |
|  |  |  |
|  |  |  |
| **HR** |  |  |
| **MAP** |  |  |
| **CVP** |  |  |
| **SV** |  |  |
| **CO** |  |  |
| **CI** |  |  |
| **SVR** |  |  |
| **SVRI** |  |  |
| **EF 4C(%)** |  |  |
| **EF 2C(%)** |  |  |
| **E/A** |  |  |
| **DT** |  |  |
| **E'** |  |  |
| **E/e'** |  |  |
| **MPI** |  |  |
